# Chemical composition and antimicrobial activity of *Satureja hortensis* and *Trachyspermum copticum* essential oil

**Published:** 2011-12

**Authors:** M Mahboubi, N Kazempour

**Affiliations:** 1 Microbiology Group, Medicinal Plants Research Center of Jundi Shapour, 87135-1178 Kashan, Iran; 2 Department of Microbiology, Research Center of Barij Essence Pharmaceutical Company, Kashan, Iran

**Keywords:** Antimicrobial activity, Thymol, *Satureja hortensis*, *Trachyspermum copticum*

## Abstract

**Background and Objectives:**

The aim of this study was to evaluate the chemical composition and antimicrobial activity of *Satureja hortensis* and *Trachyspermum copticum* essential oils against different kinds of microorganisms *in vitro*.

**Material and Methods:**

The antimicrobial activity was evaluated by micro broth dilution assay and the chemical composition of essential oils was analyzed by GC and GC/MS.

**Results:**

Thymol, p-cymene, γ-terpinene and carvacrol were the main components of *S. hortensis* oil while thymol, γ-terpinene, and o-cymene were the major components of *T. copticum* oil. Two essential oils exhibited strong antimicrobial activity but the antimicrobial activity of *T. copticum* oil was higher than that of *S. hortensis* oil.

**Conclusion:**

Thymol as a main component of oils plays an important role in antimicrobial activity.

## INTRODUCTION

During the last decade, development of antibiotic resistance as well as undesirable side effects of some drugs has led to the search for new antimicrobial agents. Many researchers have shown the plants and their essential oils have antimicrobial activity and other biological effects.

*Trachyspermum copticum* (Umbelliferae), an annual plant which grows in Iran, has white flowers and small fruits. The fruits of *T. copticum* (Ajowan) traditionally were used as diuretic, carminative, and antihelmentic (1). Some biological effects of ajowan such as antiviral (2), anti-inflammatory (3), antifungal (4), antipyretic (5), antifilarial (6), analgesic (7, 8), antinociceptive (9) and antioxidant activity (10) have been confirmed.

There are some reports on chemical composition of ajowan oil. In some reports, the major components of the oil were reported as thymol, γ-terpinene and p-cymene (4, 11, 12), and in another studies p-cymene, carvacrol and thymol were reported (13–15). Carvacrol, γ-terpinene and p-cymene were reported as main components of Iranian and African ajowan oil (16), and thymol (97.9%) was the main component of south India ajowan oil (17). Thus, four chemotypes a) thymol, γ-terpinene; b) thymol, carvacrol; c) carvacrol, γ-terpinene and d) thymol for ajowan oil have been reported so far.

*Satureja hortensis* L. (Savory) belongs to the Lamiaceae family. It has been traditionally used as stomachic, stimulant, expectorant, carminative and aphrodisiac and for treatment of different types of infectious diseases (1). Some pharmaceutical properties such as anti-diarrheal and antispasmodic (18), anti-inflammatory (19) as well as antimicrobial properties (20) were reported in the literatures.

The aim of this study was to evaluate the antimicrobial activities of ajowan and savory oils against different kinds of microorganisms and to compare their chemical composition.

## MATERIAlS AND METHODS

**Essential oil and GC/MS analysis.** The essential oils from aerial parts of *Satureja hortensis* and fruits of *Trachyspermum copticum* were prepared from Barij Essence Pharmaceutical Company, Kashan, Iran.

The oil analysis was carried out using GC and GC/MS. The GC apparatus was Agilent technology (HP) 6890 system, capillary column of HP-5MS (60 m×0.25 mm, film thickness 0.25 µm). The oven temperature program was initiated at 40°C, held for 1 min then raised up to 230°C at a rate of 3°C /min held for 10 min. Helium was used as the carrier gas at a flow rate 1.0 ml/min. The detector and injector temperatures were 250 and 230°C; respectively. GC/ MS analysis was conducted on a HP 6890 GC system coupled with 5973 network mass selective detector with a capillary column the same as above, carrier gas helium with flow rate 1 ml/min with a split ratio equal to 1/50, injector and oven temperature programmed was identical to GC. The compounds of the oil were identified by comparison of their retention indices (RI), mass spectra fragmentation with those on the stored Wiley 7 n.1 mass computer library, and NIST (National Institute of Standards and Technology) (21).

**Microbial Strains.** The microorganisms were Staphylococcus aureus ATCC 25923, Staphylococcus saprophyticus ATCC 15305, Staphylococcus epidermidis ATCC 14490, Enterococcus faecalis ATCC 29212, Enterococcus faecium ATCC 25778, Streptococcus sanguis ATCC 10556, Streptococcus salivarius ATCC 9222, Klebsiella pneumoniae ATCC 10031, Escherichia coli ATCC 8739, Salmonella typhimurium ATCC 14028, Pseudomonas aeruginosa ATCC 9027, Proteus vulgaris RI 231, Enterobacter aerogenes NCTC 10009, Shigella dysantri RI 366, Shigella flexeneri NCTC 8516, Serratia marcescens ATCC 13880, and fungi, Candida albicans ATCC 10231, Candida glabrata ATCC 90030, Aspergillus flavus, Aspergillus niger ATCC 16404, Aspergillus parasiticus ATCC 15517. Bacterial suspensions were made in Brain Heart Infusion (BHI) broth to a concentration of approximately 10^8^ CFU/ml using standard routine spectrophometrical methods.

Suspensions of fungi were made in Sabouraud dextrose broth. Subsequent dilutions were made from the above suspensions, which were then used in the tests.

**Evaluation the antimicrobial activity by micro broth dilution assay.** The minimum inhibitory concentration (MIC) and minimum lethal concentration (MLC) values of oils were determined by micro broth dilution assay. The oil was twofold serially diluted with 10% DMSO which contains 16-0.0125 µl/ml of oil. These dilutions were prepared in a 96-well microtitre plate. MOPS-buffered RPMI 1640 (fungi) (22), cation adjusted Muller Hinton broth (non fastidious bacteria) (23) and Todd Hewitt broth (fastidious bacteria) (24) were used as broth media. After shaking, 100 µl of oil was added to each well. The above microbial suspensions was diluted (1×10^6^ CFU/ml for bacteria; 10^4^ for fungi) and then 100 µl was added to each well and incubated at 35°C. MICs were defined as the lowest concentration of compound that inhibits bacteria and fungi after 24, 48 h, respectively. MLC values were the first well that showing no growth on solid media.

Thymol (45.9%), γ-terpinene (20.6%), and o-cymene (19%) were the major components of ajowan oil followed by ethylene methacrylate (6.9%), β-pinene (1.9%), and hexadecane (1.1%) (Table 1).

**Table 1:** Major components of ajowan essential oil.

Compounds	RI	(%)
α-thujene	849	0.4
α-pinene	855	0.3
β-pinene	897	1.9
β-myrcene	913	0.7
O cymene	946	19.0
β-phellendrene	948	0.4
limonene	949	0.2
γ-terpinene	984	20.6
4-terpineol	1073	0.1
cis limonene oxide	1089	0.7
dodecane	1106	0.2
β-fenchyl alcohol	1109	0.1
thymol	1200	45.9
Ethylene methacrylate	1209	6.9
Tetra decane	1316	0.2
pentadecane	1383	0.2
Diethyl phthalate	1423	0.2
hexadecane	1485	1.1
heptadecane	1571	0.1
nonadecane	1751	0.2

Thymol (28.2%), p-cymene (19.6%), γ-terpinene (16%) and carvacrol (11%) were the main components of savory oil. β-pinene (4.5%), sabinene (4.4%), α-pinene (2.7%), 4-terpineole (1.6%), and γ-terpinene were the other minor components of oil. Thymol and carvacrol consist 39.2% of total oil composition (Table 2).

**Table 2:** Major components of savory essential oil.

Compounds	RI	(%)
α-phellandrene	850	1.2
α-pinene	856	2.7
sabinene	896	4.4
β-pinene	899	4.5
β-myrcene	913	1.1
p-cymene	944	19.6
1-limonene	947	0.4
γ-terpinene	980	16.0
Cis-β-terpineol	981	0.4
Trans-pinocarveol	1037	0.3
4-terpineol	1073	1.6
p-allylanisole	1087	0.3
Cuminal	1126	0.5
carveone	1140	0.3
Trans-anethole	1163	0.6
thymol	1191	28.2
carvacrol	1198	11.0
β-elemene	1303	0.2
γ-cadinene	1380	1.5
Cis-calamenene	1382	0.3
spathulenol	1427	0.4
caryophllene oxide	1431	0.5
Tau cadinol	1498	0.7
14-norcadin-5-en-4-one isomer A	1524	0.4

The volatile oil from aerial parts of savory and that from ajowan exhibited antimicrobial activity. The MIC values of two oils were found to be at 0.06-8 µl/ml. The MIC values of ajowan and savory oils against different kinds of microorganisms were in the ranges of 0.06-16, 0.06-16 µl/ml, respectively (Table 3). Filamentous fungi and yeast were more sensitive than that of bacteria and Gram positive bacteria were less sensitive than Gram negative bacteria to both oils. The MIC values of ajowan oil against Gram positive, Gram negative bacteria and fungi were in the ranges of 0.06–4, 0.06–8, 0.025–0.5 µl/ml, respectively. The MIC values of savory oil for almost all of the microorganisms except of *Candida* species were higher than that of ajowan oil. *C. albicans* and *C. glabrata* were more sensitive to savory oil than ajowan oil or the same..

**Table 3:** The antimicrobial activity of ajowan and savory oil by micro broth dilution assay.

	Ajowan oil		Savory oil		vancomycin		Gentamicin		Amphotericin	

	MIC	MlC	MIC	MlC	MIC	MlC	MIC	MlC	MIC	MlC
***S. aureus***	1	2	2	4	1	2	–	–	–	–
***S. epidermidis***	2	4	4	8	4	8	–	–	–	–
***S. saprophyticus***	2	2	2	4	1	1	–	–	–	–
***E. faecalis***	2	4	4	4	0.5	0.5	–	–	–	–
***E. faecium***	4	4	4	8	4	4	–	–	–	–
***S. salivarius***	0.06	0.06	0.125	0.25	1	2	–	–	–	–
***S. sanjuis***	1	2	2	2	2	4	–	–	–	–
***E.coli***	0.5	0.5	1	1	–	–	2	2	–	–
***E. aerugenes***	0.5	0.5	1	1	–	–	2	4	–	–
***P. vulgaris***	0.25	0.25	0.125	0.125	–	–	1	2	–	–
***S. typhimurium***	1	1	1	1	–	–	2	4	–	–
***P. aeruginosa***	8	8	8	16	–	–	2	4	–	–
***S. marscens***	1	1	0.25	0.25	–	–	2	4	–	–
***Sh. flexeneri***	0.06	0.06	0.125	0.125	–	–	4	4	–	–
***Sh. dysantri***	0.06	0.13	0.125	0.25	–	–	2	2	–	–
***K. pneumoniae***	0.125	0.13	0.5	0.5	–	–	1	1	–	–
***C. albicans***	0.25	0.5	0.125	0.25	–	–	–	–	0.5	1
***C. glabrata***	0.25	1	0.06	0.06	–	–		–	0.5	1
***A. flavus***	0.25	0.5	0.25	0.25	–	–	–	–	8	8
***A. niger***	0.25	0.25	0.25	0.25	–	–	–	–	4	4
***A. parasiticus***	0.5	1	1	1	–	–	–	–	4	4

*S. sanguis*, S. *salivarius* and *S. aureus* were more sensitive to both oils and *Sh. flexeneri*, *Sh. dysantri* and *K. pneumoniae* were the most sensitive gram negative bacteria to both oils. Both oils showed bactericidal and fungicidal effect against microorganisms because of the same MIC and MLC values. The less sensitive microorganisms to both oils was *P. aeruginosa*.

## DISSCUSSION

Chemical composition of ajowan oil exhibited the presence of thymol, γ-terpinene and O-cymene without carvacrol as the main component of ajowan oil. *Rasooli et al*. (2008) reported that while ajowan oil contained large amount of thymol (37.2%), p-cymene (32.3%) and γ-terpinene (27.3%) (4). In addition, thymol (49%), p-cymene (15.7%) and γ-terpinene (30.8%) is reported by *Khajeh* et al. (2004) as the main components of ajowan oil (11). In the *Rasooli* et al. (2008) study, p-cymene and in other studies, γ-terpinene was the second most abundant constituent of oil. In the present study, γ-terpinene was identified as the second most common compound in ajowan oil confirming the report of *Khajeh* et al. (2004). The third most abundant compound was o-cymene. O-cymene is one of p-cymene isomers in which the alkyle group are ortho substituted. P-cymene is a naturally aromatic compound classified as monoterpene hydrocarbon consisting of a benzene ring substituted with a methyl and isopropyl groups. Ajowan oil from this study is related to the thymol, γ-terpinene chemotype.

Thymol, p-cymene, γ-terpinene and carvacrol are the main components of savory oil.

Thymol and γ-terpinene were found in two oils but the amount of thymol in ajowan oil is higher than that of savory oil and another phenolic compound of savory oil, carvacrol was not found in ajowan oil. These differences between two essential oils play an important role in their antimicrobial activities.

Antimicrobial activities of two oils are apparently attributable to high phenolic compounds such as thymol and carvacrol (25) or p-cymene, the antimicrobial effect of thymol and carvacrol is due to damage in membrane integrity with change in pH hemostasis also equilibrium of inorganic ions, p-cymene does not have antimicrobial activity but it increases the antimicrobial activity of thymol or carvacrol (26–28). P-cymene is hydrophobic compound with ability to dissolve in cytoplasmic membrane of bacterial cell between lipid acyl chains. Although the mixture of thymol and carvacrol gave additive effect (29), but the ajowan oil exhibited the higher antimicrobial activity than that of savory oil. So, other major or minor components of both oils play an important role in their antimicrobial activities. Also, the difference in sensitivity of strains to these compounds are reported (30), for example, thymol and carvacrol did not have progressive increase in antimicrobial effect against *S. thyphimurium* or the antimicrobial effect of thymol toward *E. coli*, *S. thyphimurium*, *S. aureus*, *B. subtilis* was higher than that of p-cymene at the same concentration (31). The antimicrobial activity of thymol, carvacrol, p-cymene and γ-terpinene against *S. aureus* and *E. coli* is reported by Cristani et al. (2007) (32). Among four compounds thymol is more toxic against *S. aureus* than the other three components, while carvacrol and p-cymene are the best inhibitory against *E. coli*. γ-terpinene, the non oxygenated hydrocarbon was active against *S. aureus*, *C. albicans* but did not inhibit the growth of *P. aeruginosa*, *E. coli* (33) and *S. thyphimurium* (34). γ-terpinene has been reported to be inactive in broth microdilution assay (35) and agar dilution assay (36). γ-terpinene and p-cymene are inactive against *P. aeruginosa* and they are unable to penetrate the outer membrane. Non-oxygenated monoterpene hydrocarbons such as γ-terpinene and p-cymene appear to produce antagonistic effects against more tolerant micro-organisms such as *P. aeruginosa* (33). For these reasons, yeast and Gram negative bacteria are more sensitive to both oils especially toward ajown oil and *P. aeruginosa* is less sensitive organism to both of them.

The high p-cymene content of essential oil has antagonized the antimicrobial action of phenol, resulting in a weaker activity of oil (37). O-cymene has been found to be present in the essential oil from *Pteronia incana* (38), *Eriosema englerianum* (39) and *Diplotaenia damavandica* the later oil showed strong antimicrobial activities against *S. aureus*, *B. subtilis*, *S. epidermidis* and *E. coli* (40). O-cymene has antifungal activity (39). It is concluded that the presence of thymol as major component of two oils plays an important role in antimicrobial activity of oils and the mixture of different major components with each other in essential oils have important role in their antimicrobial effect. It is found that the whole essential oils have a greater antibacterial activity than the mixed major component (41) so the minor components of essential oil play a critical role for activity of oil. α-pinene was found to have the antimicrobial activity against *S. aureus*, *S. epidermidis*, *propionibacterium acnes* (42), *C. albicans* (43), mold and pathogenic yeast (44). It disrupted the cytoplasmic membrane of yeast and Gram positive bacteria and inhibited respiratory activity in yeast mitochondria (45). β-pinene had antifungal activity (46). β-myrcene like p-cymene did not exhibit considerable antimicrobial effect but it enhanced the activities of other components in whole essential oil (47). Gram negative bacteria usually are less susceptible to essential oil than Gram positive bacteria because of the outer membrane protein which restricts diffusion of compounds through it (48), while our results did not exhibit any selectivity toward Gram positive bacteria and Gram negative bacteria except of *P. aeruginosa* that is sensitive to both oils than gram positive bacteria. In conclusion, thymol is important antimicrobial agents in essential oils.

Further *in vivo* experiments are obviously needed to demonstrate the therapeutic values of ajowan and savory oils. As we showed two oils were highly effective against a broad spectrum of microorganisms.
